# Supplementary figures and images for: Systematic interrogation of diverse Omic data reveals interpretable, robust, and generalizable transcriptomic features of clinically successful therapeutic targets
Source: PLoS Comput Biol. 2018 May 21;14(5):e1006142. doi: 10.1371/journal.pcbi.1006142 (PMC5983857; doi:10.1371/journal.pcbi.1006142)

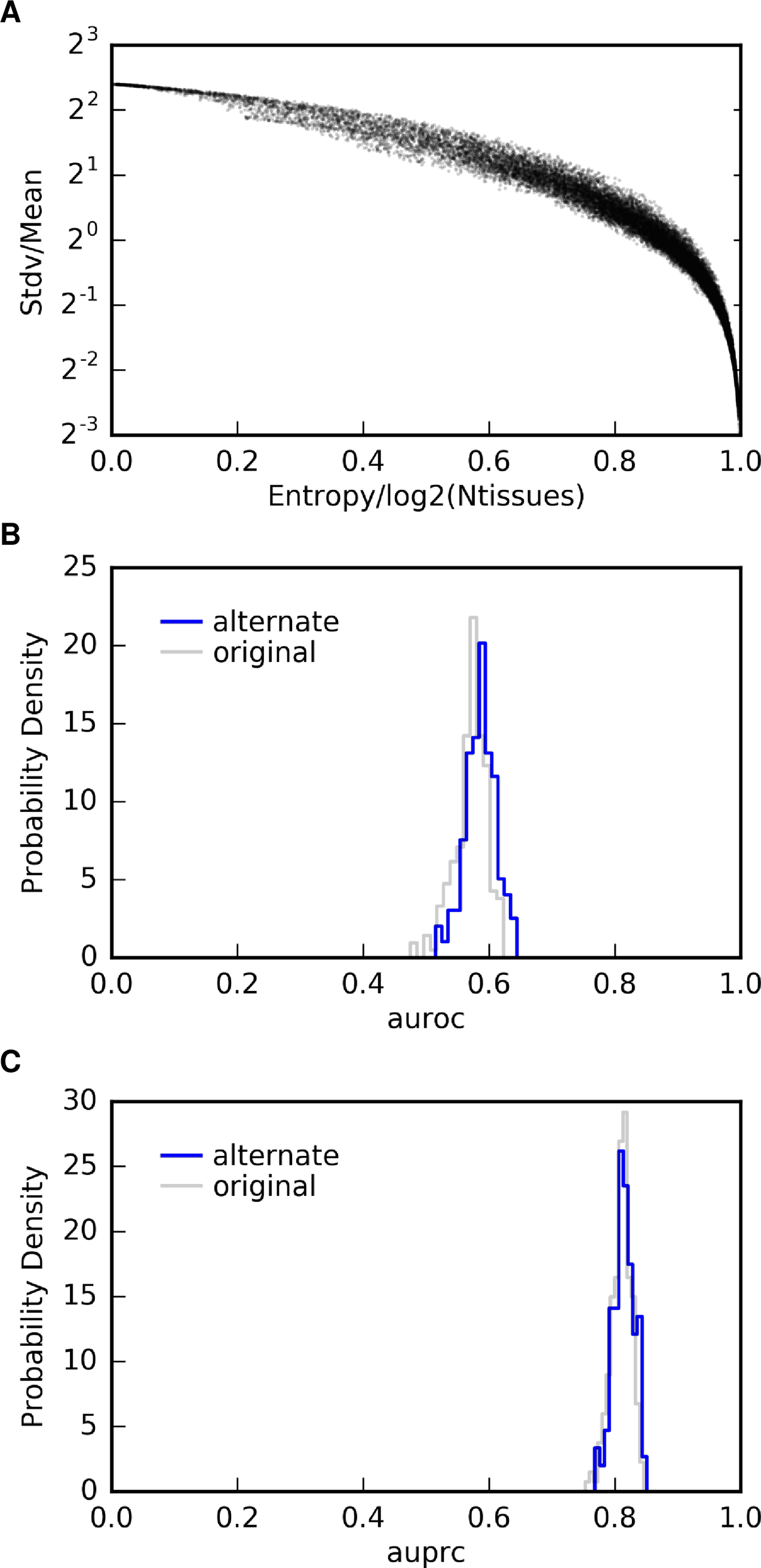

Supplement: S1 Fig — (A) Relationship between coefficient of variation (standard deviation/mean) of gene expression across tissues and entropy of gene expression across tissues. Entropy of a target was defined as sum[Pi log2(1/Pi)] where Pi = Ei/sum(Ei) and Ei is the target’s expression in the ith tissue). Coefficient of variation and entropy were computed using un-log-transformed expression values. The strong (nonlinear) correlation indicates that entropy captures similar information about the distribution of a target’s expression across tissues as the pair of mean and standard deviation. (B) Distribution of area under the receiver operating characteristic curve (AUROC) values from 200 repetitions of 5-fold cross-validation. The light gray distribution corresponds to the original analysis that included the mean and standard deviation of gene expression across tissues as candidate target features. The dark blue distribution corresponds to the alternative analysis that replaced the mean and standard deviation features with entropy of gene expression across tissues as a candidate target feature. The models had nearly identical AUROC distributions. (C) Distribution of area under the precision-recall curve (AUPRC) values from 200 repetitions of 5-fold cross-validation. The models had nearly identical AUPRC distributions. (TIF) [file pcbi.1006142.s009.tif]

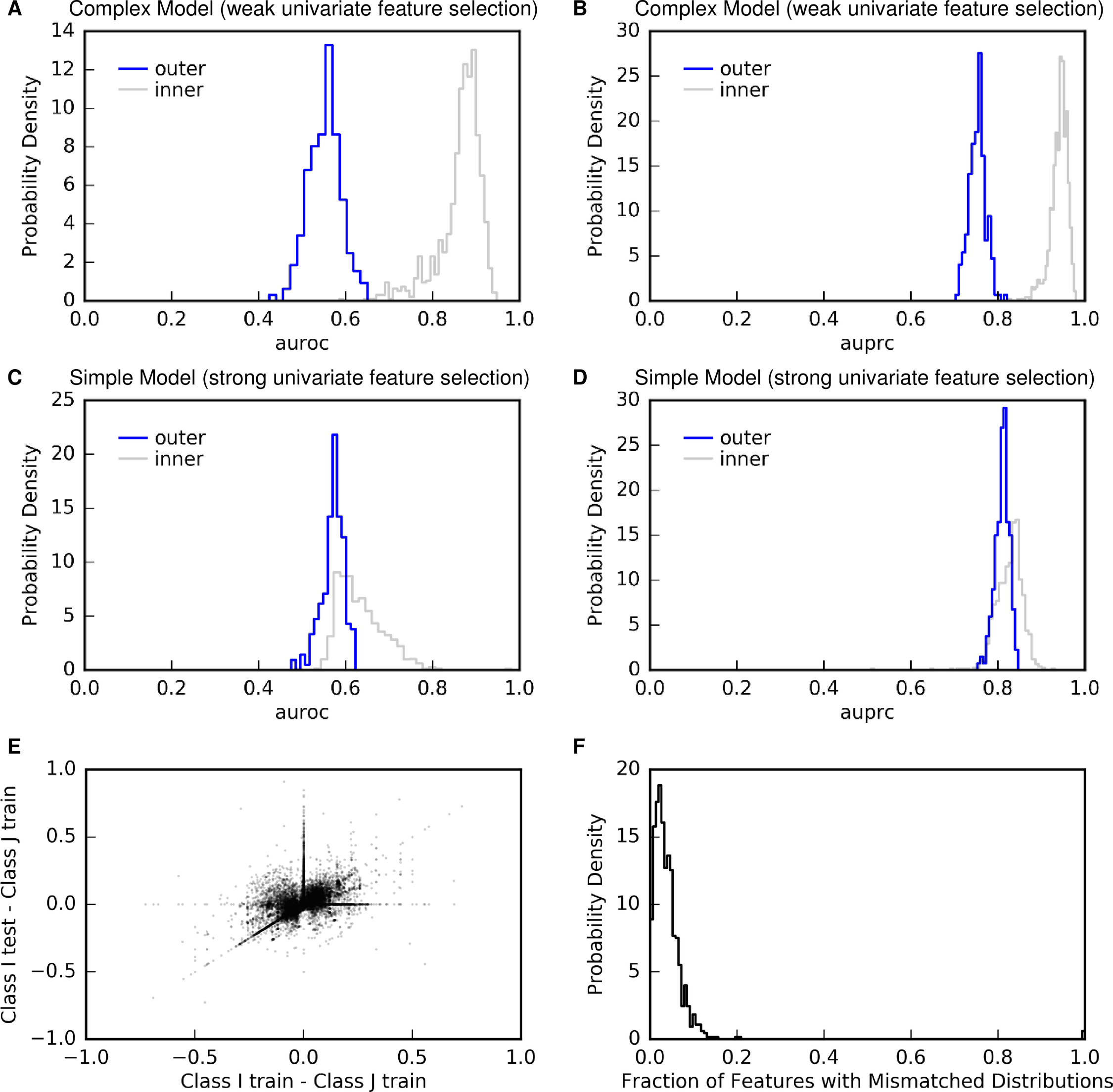

Supplement: S2 Fig — (A) Distribution of area under the receiver operating characteristic curve (AUROC) values from the alternative modeling pipeline with weak univariate feature selection (nominal p-value less than 0.05), which allowed hundreds of features to pass through to the multivariate feature selection step (Random Forest feature ranking with incremental feature elimination). The light gray distribution corresponds to the inner cross-validation loop performance of the model. The dark blue distribution corresponds to the outer cross-validation loop performance of the model. There is a large discrepancy between the distributions, indicating failure of the inner cross-validation loop to appropriately tune the complexity of the model. (B) Distribution of area under the precision-recall curve (AUPRC) values from the alternative modeling pipeline with weak univariate feature selection. (C and D) Distribution of AUROC and AUPRC values from the original modeling pipeline with strong univariate feature selection (multiple hypothesis testing corrected p-value less than 0.05). There is little discrepancy between generalization performance estimated by the inner and outer cross-validation loops, indicating appropriate tuning of the complexity of the model. (E) Illustration of the mismatch between training and testing examples that arises from splitting our small and heterogeneous sample of targets. Each point in the scatterplot corresponds to a feature that passed through the weak univariate feature selection step of the alternative modeling pipeline. Plotted on the horizontal axis is the difference between the median of the Class I training examples and the median of the Class J training examples, where if Class I is success, then Class J is failure, and vice versa. Plotted on the vertical axis is the difference between the median of the Class I TESTING examples and the median of the Class J training examples. For features in the first quadrant, the Class I training and testing examples bo [file pcbi.1006142.s010.tif]
