# Supplementary material for: Systematic interrogation of diverse Omic data reveals interpretable, robust, and generalizable transcriptomic features of clinically successful therapeutic targets
Source: PLoS Comput Biol. 2018 May 21;14(5):e1006142. doi: 10.1371/journal.pcbi.1006142 (PMC5983857; doi:10.1371/journal.pcbi.1006142)
Supplement: S1 Text — (DOCX) [file pcbi.1006142.s011.docx]

**S1 Text.** Description of analysis of gene expression entropy across tissues as a feature quantifying tissue specificity of target expression.

To further investigate the hypothesis that tissue specific expression is a favorable target feature, we re-ran the entire modeling pipeline (Fig 2) with gene expression entropy, a feature explicitly quantifying specificity of gene expression across tissues ([Yao and Rzhetsky. Quantitative systems-level determinants of human genes targeted by successful drugs. Genome Res. 2008](https://www.ncbi.nlm.nih.gov/pubmed/18083776)), appended to each tissue expression dataset instead of the mean and standard deviation features.

Entropy is equal to zero bits if the target is expressed in a single tissue and reaches a maximum of log2(number of tissues) bits if the target is expressed uniformly across tissues. This is arguably the best way to quantify tissue specificity in a single feature. Also, note that entropy is highly (nonlinearly) correlated with the coefficient of variation (standard deviation divided by mean) across tissues (S1 Fig A). If tissue specificity is the predictive signal in the mean and standard deviation features, then entropy should perform as well as the mean and standard deviation pair. To fairly compare the entropy feature to all features previously evaluated, we re-ran the entire model building pipeline (Fig 2) with the entropy feature appended to each tissue expression dataset instead of the mean and standard deviation features.

The entropy feature was selected 610 times out of 1000 train-test cycles and was the single feature in 298 of 599 single feature models (S6 Table). Model performance was nearly identical to the performance of the original model, with a median AUROC of 0.59 and median AUPRC of 0.81 (S1 Fig B and C). Even though the mean feature was not explicitly included in this analysis, during dimensionality reduction, large groups of tissue expression features were averaged together, forming surrogate mean features. These surrogate mean features were selected 381 times out of 1000 train-test cycles, appearing as the single selected feature 299 times. A mean or entropy feature appeared 991 times, but the two features never appeared together. These results support the hypothesis that tissue specific target expression as measured by entropy is predictive of target success, but do not rule out mean tissue expression as an alternative predictor. A better analysis of the tissue specificity hypothesis would be to examine target-indication pairs and evaluate whether the target is specifically expressed in the tissue relevant for the indication treated in each clinical trial.
