# Supplementary material for: Systematic interrogation of diverse Omic data reveals interpretable, robust, and generalizable transcriptomic features of clinically successful therapeutic targets
Source: PLoS Comput Biol. 2018 May 21;14(5):e1006142. doi: 10.1371/journal.pcbi.1006142 (PMC5983857; doi:10.1371/journal.pcbi.1006142)
Supplement: S2 Text — (DOCX) [file pcbi.1006142.s012.docx]

**S2 Text.** Description of analysis of multivariate feature selection (modeling pipeline with less stringent univariate feature selection).

We employed a stringent univariate feature selection step (Fig 2, Step 2) in our modeling pipeline, which diminished the chance of the multivariate feature selection step (Fig 2, Step 4) finding highly predictive combinations of features that individually were insignificantly predictive.

We employed univariate feature selection to intentionally bias the analysis toward simple, interpretable models. Also, we assumed *a priori* that most features would be uninformative and therefore univariate feature selection served to reduce the computational demand of the analysis. Furthermore, there is a practical issue that arises because datasets have incomplete and only partially overlapping coverage of targets. Consequently, concatenating datasets to pass all features into a multivariate feature selection step would yield a feature matrix with huge blocks of missing values. Finally, the sample of targets with phase III clinical trial outcomes is small (259 successes and 72 failures), leading to a non-negligible probability that, given hundreds or thousands of candidate features, by chance there will be combinations of features that separate training data well but generalize poorly ([Rubingh et al. Assessing the performance of statistical validation tools for megavariate metabolomics data.](https://www.ncbi.nlm.nih.gov/pubmed/24489531) *[Metabolomics](https://www.ncbi.nlm.nih.gov/pubmed/24489531)*[. 2006](https://www.ncbi.nlm.nih.gov/pubmed/24489531); [Cawley and Talbot. On Over-fitting in Model Selection and Subsequent Selection Bias in Performance Evaluation. *Journal of Machine Learning Research*. 2010](http://www.jmlr.org/papers/v11/cawley10a.html)).

As a compromise, we re-ran the entire model building pipeline (Fig 2) with the threshold for the univariate feature selection step made less stringent by eliminating the multiple hypothesis testing correction and accepting features with nominal p-values less than 0.05. This allowed hundreds of features to pass through to the multivariate feature selection step (Random Forest with incremental feature elimination) and ultimately dozens of features (median of 73) were selected for each of the final models in the 1000 train-test cycles (S6 Table). Despite this increase in number of features, the mean expression and standard deviation of expression features were still robustly selected, appearing in 958 and 745 models, respectively. Although the models performed exceptionally well on the inner cross-validation loop, with a median AUROC of 0.88 and median AUPRC of 0.94, they performed no better than the simple models on the outer cross-validation loop, with a median AUROC of 0.56 and median AUPRC of 0.75 (S2 Fig A-D).

The large discrepancy between inner and outer cross-validation performance can be explained by a mismatch between training and testing distributions that arises because the sample size is small and the diversity of samples is large (for example, 71 gene families are represented in the set of 331 targets). When we perform an 80:20 split of the data, there is a good chance that the targets in the testing set will have characteristics that differ from the targets in the training set. The inner cross-validation loop fails to tune the model to an appropriate (low) complexity and over-estimates the generalization performance of the model because model tuning by cross-validation depends on subsets of the training examples being representative of future test examples. For our small and heterogeneous sample of targets, this requirement does not appear to be satisfied, and other means of limiting model complexity are needed, such as stricter univariate feature selection.

To test for features with a mismatch between training and testing distributions, we compared two differences: 1) the median of the successful targets in the training set minus the median of the failed targets in the training set, and 2) the median of the successful targets in the TESTING set minus the median of the failed targets in the training set. If these two differences have opposite sign, it means the successful targets in the TESTING set will be on the wrong side of a decision boundary separating the successful and failed targets in the training set. In a similar way, by swapping successful and failed targets in the difference calculations, we also checked for features where failed targets in the testing set will be on the wrong side of a decision boundary learned from the training data. Many features exhibit mismatch, which explains the large discrepancy between inner and outer cross-validation performance (S2 Fig E and F). Note that as the magnitude of the difference between successful and failed targets in the training set becomes large, mismatch features (in the second and fourth quadrants) become scarce while robust features (in the first and third quadrants) remain. This is analogous to increasing the stringency of univariate feature selection. Also, note that this analysis only checked for mismatches of individual features. Many more mismatches can occur with feature combinations. In summary, predictive feature combinations may exist, but our sample size is not large enough to robustly identify which of all apparently predictive feature combinations will generalize well.
